# Graphene perfect absorber of ultra-wide bandwidth based on wavelength-insensitive phase matching in prism coupling

**DOI:** 10.1038/s41598-019-48501-w

**Published:** 2019-08-19

**Authors:** Sangjun Lee, Hyungjun Heo, Sangin Kim

**Affiliations:** Department of Electrical and Computer Engineering, Ajou University, Suwon, South Korea

**Keywords:** Optical properties and devices, Optoelectronic devices and components

## Abstract

We proposed perfect absorbers of ultra-wide bandwidths based on prism coupling with wavelength-insensitive phase matching, which consists of three dielectric layers (Prism-Cavity-Air) with monolayer graphene embedded in the cavity layer. Due to inherent material dispersion of the dielectric layers, with the proper choice of the incidence angle and the cavity thickness, the proposed perfect absorbers can satisfy the phase matching condition over a wide wavelength range, inducing enormous enhancement of the absorption bandwidth. The requirement on the material dispersions of the prism and the cavity layer for the wavelength-insensitive phase matching over a wavelength range of the interest has been derived, and it has been demonstrated that the various kinds of materials can meet the requirement. Our theoretical investigation with the transfer matrix method (TMM) has revealed that a 99% absorption bandwidth of ~300 nm with perfect absorption at *λ*  = 1.51 μm can be achieved when BK7 and PDMS are used as the prism and the cavity layer, respectively, which is ~7 times wider than the conceptual design based on the non-dispersive materials. The full width at half maximum of our designed perfect absorber is larger than 1.5 μm.

## Introduction

Due to high carrier mobility and uniform light absorption over a wideband wavelength range from visible to terahertz, the graphene has attracted enormous interests in developing high-speed photodetectors^1–6^. Absorption efficiency of ~2.3% is relatively high value despite of atomically thin graphene of ~0.34 nm. However, for practical high-performance photodetectors, the absorption should be enhanced greatly by applying the resonant structures such as gratings, photonic crystals, and prism couplers^7–25^.

In this work, we focus on the remarkable enlargement of absorption bandwidth in the graphene perfect absorbers based on prism coupling^16–25^. Except that the substrate layer is replaced with air, the considered absorber is basically quite similar to the structures reported in refs^16,17^, in which the monolayer graphene is embedded in the cavity layer between prism and substrate to enable practical perfect absorption with naturally available materials. At the incidence angle of interest with total internal reflection (TIR) at the bottom of the cavity layer, the proposed structure can be considered as a one-port resonant system. From the coupled mode theory (CMT), the absorption in a lossy resonator is given as A = 4*γ*_leak_*γ*_loss_/[(*ω* − *ω*_o_)^2^ + (*γ*_leak_ + *γ*_loss_)^2^], where *ω*_o_, *γ*_leak_, and *γ*_loss_ are a resonant frequency, a leakage rate, and a loss rate, respectively^13,16^. The perfect absorption is achieved at the resonance frequency (*ω* = *ω*_o_) when the critical coupling condition (*γ*_loss_ = *γ*_leak_) is satisfied, and the full width at half maximum (FWHM) of the perfect absorber is given by 4*γ*_loss_. Obviously, the bandwidth of the perfect absorber can be increased with a larger loss rate, which, however, is not an option in this work since atomically thin monolayer graphene is the lossy medium. In the prism coupling structure^16–25^, the resonance frequency (or wavelength) is determined by the phase matching among multiple reflected waves, and the phase retardation in the cavity layer depends on an incidence angle. So, the resonance wavelength also depends on the incidence angle; for a given incidence angle, the phase matching is satisfied only at a specific wavelength, where the perfect absorption can be achieved under the critical coupling condition.

As an entirely new scheme to increase the bandwidth of the perfect absorber, manipulation of the resonance condition has been considered here. If the prism coupling absorber structure is designed so as to satisfy the phase matching over a wide range of wavelength, the almost perfect absorption can be achieved over a wide wavelength range, simultaneously increasing the FWHM. Our investigation has revealed that almost wavelength-insensitive phase matching can be achieved over a wide wavelength range with a proper choice of the prism and the cavity layer materials, where the material dispersions (d*n*/d*λ*≠0) compensate for the wavelength-dependent phase retardation variation. Our theoretical study has shown that almost perfect absorption (>99%) can be obtained over a wavelength range of ~300 nm with a choice of naturally available materials for the prism and the cavity layer, which is the best as far as we know. The proposed absorption scheme based on the wavelength-insensitive phase matching will be also useful for applications such as optical sensors^23–25^, solar cells^26^, thermal emitters^27^, and nonlinear optics^28^.

## Results

### Principle of the wavelength-insensitive phase matching

**Figure 1:**
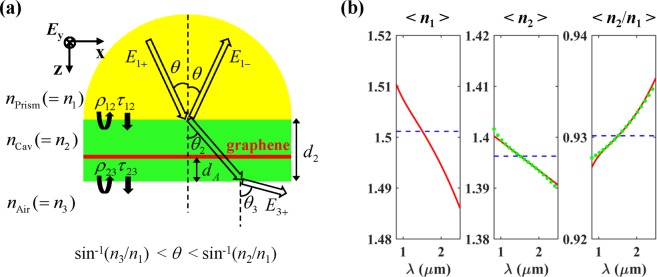
(**a**) Schematic of the proposed graphene perfect absorber composed of Prism(*n*_1_)-Cavity(*n*_2_)-Air(*n*_3_), where monolayer graphene is embedded in the cavity and *n*_1_ > *n*_2_ > *n*_3_ = 1. The red thin layer represents the monolayer graphene of 0.34 nm thickness as an absorbing medium. (**b**) The material dispersions of BK7 glass (*n*_1_) and PDMS (*n*_2_), and the ratio (*n*_2_/*n*_1_) between them over the wavelength range of *λ* = 0.82~2.50 μm. The blue, red, and green curves represent the cases of non-dispersion, real dispersion, and ideal dispersion, respectively.

The proposed absorber (Fig. 1(a)) consists of a prism (*n*_1_), a cavity layer (*n*_2_), air (*n*_3_), and monolayer graphene embedded in the cavity layer, where *n*_1_ > *n*_2_ > *n*_3_ = 1. For the permittivity of graphene, Kubo formulation was used with parameters of a graphene thickness of 0.34 nm, Fermi level of 0 eV (undoped), Fermi velocity of 10^6^ m/s, and mobility of 0.5 m^2^/Vs^29,30^. In this work, BK7 glass and PDMS are used as the prism and the cavity layer, respectively, unless otherwise stated. Like most transparent materials, BK7 and PDMS have ‘*normal dispersion*’, that is, their refractive indices monotonically decrease with wavelength (d*n*/d*λ* < 0), as represented by red curves in Fig. 1(b) ^31,32^. It is noteworthy that the index ratio of *n*_2_/*n*_1_ is proportional to wavelength, which is the basic requirement for wavelength-insensitive phase matching, as will be discussed later. We considered transverse electric (TE, or s-polarized) wave illumination from the prism with an incidence angle of *θ*, whose electric field is perpendicular to the incidence plane. All the reflection and absorption calculations were conducted using the transfer matrix method (TMM)^33,34^.

The proposed structure based on prism coupling can be considered as a one-port resonant system for a certain range of incidence angle, sin^−1^ (*n*_3_/*n*_1_) < *θ* < sin^−1^ (*n*_2_/*n*_1_), where TIR occurs at the cavity-air interface while partial reflection occurs at the prism-cavity interface. If multiple reflected waves interfere destructively at the prism-cavity interface and thus, total reflection becomes zero, perfect absorption is achieved. The perfect absorption is a result of strong resonance, at which the strongly confined light forms a standing wave in the cavity layer. As mentioned earlier, according to the CMT, the resonance condition (*ω* = *ω*_o_) and the critical coupling condition (*γ*_loss_ = *γ*_leak_) are simultaneously required to obtain the perfect absorption. The loss rate can be controlled by the graphene position (*d*_A_) for a given absorber structure with a specific leakage rate, as demonstrated already in ref. ^16^. However, the graphene position almost does not affect the resonance condition because graphene is too thin (0.34 nm)^16,17^. So, the resonance condition enabling the absorption peak is mainly determined by the dielectric multilayer structures (prism-cavity-air) without graphene. We confirmed that if the graphene layer is removed under the perfect absorption condition, the reflection coefficient (*r*) becomes approximately −1 (Supplementary Information). The slight difference from *r* = −1 stems from the phase retardation due to the graphene layer, which is negligibly small as aforementioned.

Using the TMM, the resonance condition of *r* = −1 can be rearranged into (Supplementary Information).

1a $$exp({\rm{j}}2{\delta }_{2})=-\,{\rho }_{23},$$

and

1b $$2{\delta }_{2}=4{\rm{\pi }}{d}_{2}{n}_{2}\,\cos \,{\theta }_{2}/{\rm{\lambda }}=4{\rm{\pi }}{d}_{2}\sqrt{{n}_{2}^{2}-{n}_{1}^{2}si{n}^{2}\theta }/{\rm{\lambda }},$$

where 2*δ*_2_ is the phase retardation experienced by the wave during a round trip in the cavity layer, and *ρ*_23_ is the Fresnel reflection coefficient at the cavity-air interface, which is given by

2 $${\rho }_{23}=({n}_{2}cos{\theta }_{2}-{n}_{3}cos{\theta }_{3})/({n}_{2}cos{\theta }_{2}+{n}_{3}cos{\theta }_{3}).$$

The phase retardation is affected by both the refractive indices of *n*_1_ and *n*_2_. Under the constraint of sin^−1^ (*n*_3_/*n*_1_) < *θ* < sin^−1^ (*n*_2_/*n*_1_), the phase matching condition for the resonance is expressed by 2*δ*_2_ − *ϕ* = 2π*m*, where *m* is an integer and *ϕ* is the phase of −*ρ*_23_, because the amplitude of *ρ*_23_ is unity. By rearranging the phase matching condition, we obtain

3 $${({n}_{2}/{n}_{1})}^{2}={\sin }^{2}\theta +{(\lambda /{n}_{1})}^{2}{(\varphi +2\pi m)}^{2}/{(4\pi {d}_{2})}^{2}.$$

**Figure 2:**
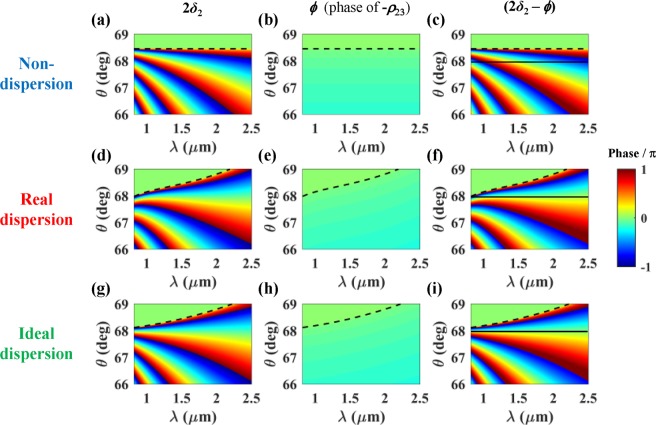
Phase retardation experienced by the wave during a round trip in the cavity layer of *d*_2_ = 6.4 μm (2*δ*_2_), *ϕ* (the phase of −*ρ*_23_), and phase difference (2*δ*_2_ − *ϕ*) as a function of wavelength and incidence angle for non-dispersion (top panel), real dispersion (middle panel), and ideal dispersion (bottom panel) of the prism (BK7) and cavity layers (PDMS). The black dashed lines correspond to critical angles at the prism-cavity interface. The black solid lines in (**c**,**f**,**i**) correspond to *θ* = 67.98 deg. Note that the phase analysis was conducted without the graphene layer.

As mentioned earlier, for broadband absorption, the wavelength-insensitive phase matching is required, which implies (3) is satisfied over a wide wavelength range for given values of *θ*, *m*, and *d*_2_. It is obvious that this requirement cannot be met if the prism and the cavity layer materials are non-dispersive, that is, d*n*_1_/d*λ*  = d*n*_2_/d*λ*  = 0. In (2), since cos*θ*_3_ is pure imaginary and |*n*_3_cos*θ*_3_| is much larger than |*n*_2_cos*θ*_2_| when sin^−1^ (*n*_3_/*n*_1_) < *θ* < sin^−1^ (*n*_2_/*n*_1_), *ϕ* is quite close to zero and almost constant over a wide wavelength range even for the dispersive prism and cavity. The validity of this will be demonstrated later for the practical materials (Fig. 2). Then, assuming the materials of normal dispersion for the prism and the cavity layer (d*n*/d*λ* < 0), the right-hand side of (3) is a monotonically increasing function of *λ* for any given *θ*, *m*, and *d*_2_. So, if *n*_2_/*n*_1_ is an increasing function of *λ*, there is a chance to satisfy (3) approximately over a wide wavelength range as discussed in detail below. To have d(*n*_2_/*n*_1_)/d*λ* > 0, that is, *n*_2_/*n*_1_ an increasing function of *λ*, it is required that (*n*_2_/*n*_1_)d*n*_1_/d*λ* < d*n*_2_/d*λ* < 0. This means that the index of the prism should decrease faster than that of the cavity as *λ* increases, that is, |d*n*_1_/d*λ*| > |d*n*_2_/d*λ*| to obtain the wavelength-insensitive phase matching.

From Sellmeier’s equation, refractive indices of dielectric materials are modeled as

4 $${n}^{2}=1+\frac{{A}_{1}{\lambda }^{2}}{{\lambda }^{2}-{\lambda }_{1}^{2}}+\frac{{A}_{2}{\lambda }^{2}}{{\lambda }^{2}-{\lambda }_{2}^{2}}+\frac{{A}_{3}{\lambda }^{2}}{{\lambda }^{2}-{\lambda }_{3}^{2}},$$

where *A*_i_ and *λ*_i_ are the fitting coefficients and the characteristic wavelengths, respectively^31,35^. For most of dielectric materials which are transparent in visible and near infrared wavelength ranges, *λ*_1_ and *λ*_2_ are around or smaller than 0.1 μm, and λ_3_ is around 10 μm^31,35^. So, for the wavelength range of our interest (~1 μm < *λ* < ~2.5 μm), we can consider that *λ*_1_, *λ*_2_ ≪ *λ* ≪ *λ*_3_, and thus, (4) can be roughly approximated as

5 $${n}^{2}\approx 1+{A}_{1}+{A}_{2}-\frac{{A}_{3}{\lambda }^{2}}{{\lambda }_{3}^{2}}={{n}_{0}}^{2}-\frac{{A}_{3}{\lambda }^{2}}{{\lambda }_{3}^{2}}.$$

Then, the index ratio for two dielectric materials can also be approximated as

6 $${(\frac{{n}_{2}}{{n}_{1}})}^{2}=\frac{{{n}_{02}}^{2}-\frac{{A}_{32}{\lambda }^{2}}{{\lambda }_{32}^{2}}}{{{n}_{01}}^{2}-\frac{{A}_{31}{\lambda }^{2}}{{\lambda }_{31}^{2}}}\approx \frac{{{n}_{02}}^{2}}{{{n}_{01}}^{2}}(1+(\frac{{A}_{31}}{{{n}_{01}}^{2}{\lambda }_{31}^{2}}-\frac{{A}_{32}}{{{n}_{02}}^{2}{\lambda }_{32}^{2}}){\lambda }^{2}),$$

where *n*_0j_, *A*_3i_, *λ*_3j_ are the elements of *n*_j_ in (5). Using (6), (3) becomes

7 $${{n}_{01}}^{2}{\sin }^{2}\theta -{{n}_{02}}^{2}+({(\frac{\varphi +2\pi m}{4\pi {d}_{2}})}^{2}-\frac{{A}_{31}}{{\lambda }_{31}^{2}}{\sin }^{2}\theta +\frac{{A}_{32}}{{\lambda }_{32}^{2}}){\lambda }^{2}=0.$$

Therefore, we can achieve the phase matching over a wide wavelength range of our interest by choosing *m*, *θ*, and *d*_2_ properly as far as *n*_01_ > *n*_02_, ${n}_{02}^{2}/{n}_{01}^{2} > ({A}_{32}/{\lambda }_{32}^{2})/({A}_{31}/{\lambda }_{31}^{2})$ and (*λ*_11_, *λ*_21_ *λ*_12_, *λ*_22_) ≪*λ* ≪ (*λ*_31_, *λ*_32_). Under the constraint, the coefficient of *λ*^2^ in (6) becomes positive and thus, *n*_2_/*n*_1_ is a monotonically increasing function.

Based on this, BK7 and PDMS were chosen as the materials for the prism and the cavity layer, respectively. In Fig. 1(b) (the red curves), one can see that they meet the requirement on the material dispersions over a wide wavelength range. For the chosen materials, we have investigated the possibility of the wavelength-insensitive phase matching in the prism coupling structure without graphene. For the real indices of BK7 and PDMS, the optimal *θ* = 67.98 deg and *d*_2_ = 6.4 μm were found to satisfy (3) as close as possible with *m* = 1 over a wide wavelength range. Figure 2 shows 2*δ*_2_, *ϕ*, and their difference (2*δ*_2_ − *ϕ*) as a function of wavelength and incidence angle with *d*_2_ = 6.4 μm for three different cases of dispersion: the non-dispersion (constant index), real dispersion, and ideal dispersion (artificial cavity material).

For the non-dispersion case, the indices of the prism and the cavity were set to be *n*_1_ = 1.5012 and *n*_2_ = 1.3963, respectively, corresponding to the index values at *λ* = 1.51 μm in the real dispersions as represented by the blue curves in Fig. 1(b). As shown in Fig. 2(a–c), for any incidence angle below the critical angle of sin^−1^ (*n*_2_/*n*_1_) = 68.4539 deg, *ϕ* is almost constant, and thus, both 2*δ*_2_ and (2*δ*_2_ − *ϕ*) decrease with *λ* as expected from (1b), so that the wavelength-insensitive phase matching cannot be achieved. Regardless of the cavity thickness, this tendency remains as expected from (3).

For the real dispersion case, the real refractive indices of BK7 and PDMS represented by the red curves in Fig. 1(b) were used. Similar to the non-dispersion case, *ϕ* is almost constant and close to zero as aforementioned (Fig. 2(e)). Interestingly, depending on the range of *θ*, 2*δ*_2_ and (2*δ*_2_ − *ϕ*) show three different *λ* dependences (Fig. 2(d,f)). When *θ* is above (below) the optimal incidence angle of 67.98 deg, they increase (decrease). Whereas, around the optimal incidence angle, they show very weak *λ* dependence, remaining close to zero (equivalent to 2 *mπ*) in the wide wavelength range of ~1.3 μm < *λ* < ~2.0 μm. This implies that the wavelength-insensitive phase matching is achieved for the chosen optimal values of *θ* and *d*_2_ because BK7 and PDMS meet the requirement on the material dispersions over the corresponding wavelength range. All these *λ* dependences of 2*δ*_2_ can be understood in terms of the increment rate of $\sqrt{{n}_{2}^{2}-{n}_{1}^{2}si{n}^{2}\theta }$ relative to *λ* in (1b), which is directly related to the *λ* dependences of (*n*_2_/*n*_1_)^2^ and the critical angle at the prism-cavity interface (sin^−1^ (*n*_2_/*n*_1_)). It is noteworthy that the uppermost parts of the equi-phase contour lines for 2*δ*_2_ closely follow the critical angle curves which are represented by the black dashed lines in Fig. 2. This provides another intuitive understanding of the wavelength-insensitive phase matching: it stems from the fact that the critical angle at the top interface of the proposed structure increases with *λ*, which requires that *n*_2_/*n*_1_ is an increasing function of *λ*.

In order to verify the wavelength-insensitive phase matching performance with BK7 and PDMS, we considered artificial cavity material whose dispersion can ideally satisfy (3) for the prism made of real BK7 with the same optimal* θ* and *d*_2_. The green curves in Fig. 1(b) represent the index of the ideal cavity material (*n*_2_) extracted from (3) with *m* = 1, *θ* = 67.98 deg, and *d*_2_ = 6.4 μm, and its ratio to the index of BK7 (*n*_2_/*n*_1_). One can see that the green curves are very close to the red (real) ones in both cases of *n*_2_ and *n*_2_/*n*_1_. This means that the performance of the wavelength-insensitive phase matching with BK7 and PDMS is close to the ideal case. As shown in Fig. 2(g–i), the wavelength dependence of 2*δ*_2_, *ϕ*, and (2*δ*_2_ − *ϕ*) with ideal *n*_2_ is very similar to the real dispersion case. The only difference is that the phase matching is definitely independent of wavelength for the optimal incidence angle (*θ* = 67.98 deg), as seen in the black solid line in Fig. 2(i).

### Ultra-wideband absorption based on wavelength-insensitive phase matching

**Figure 3:**
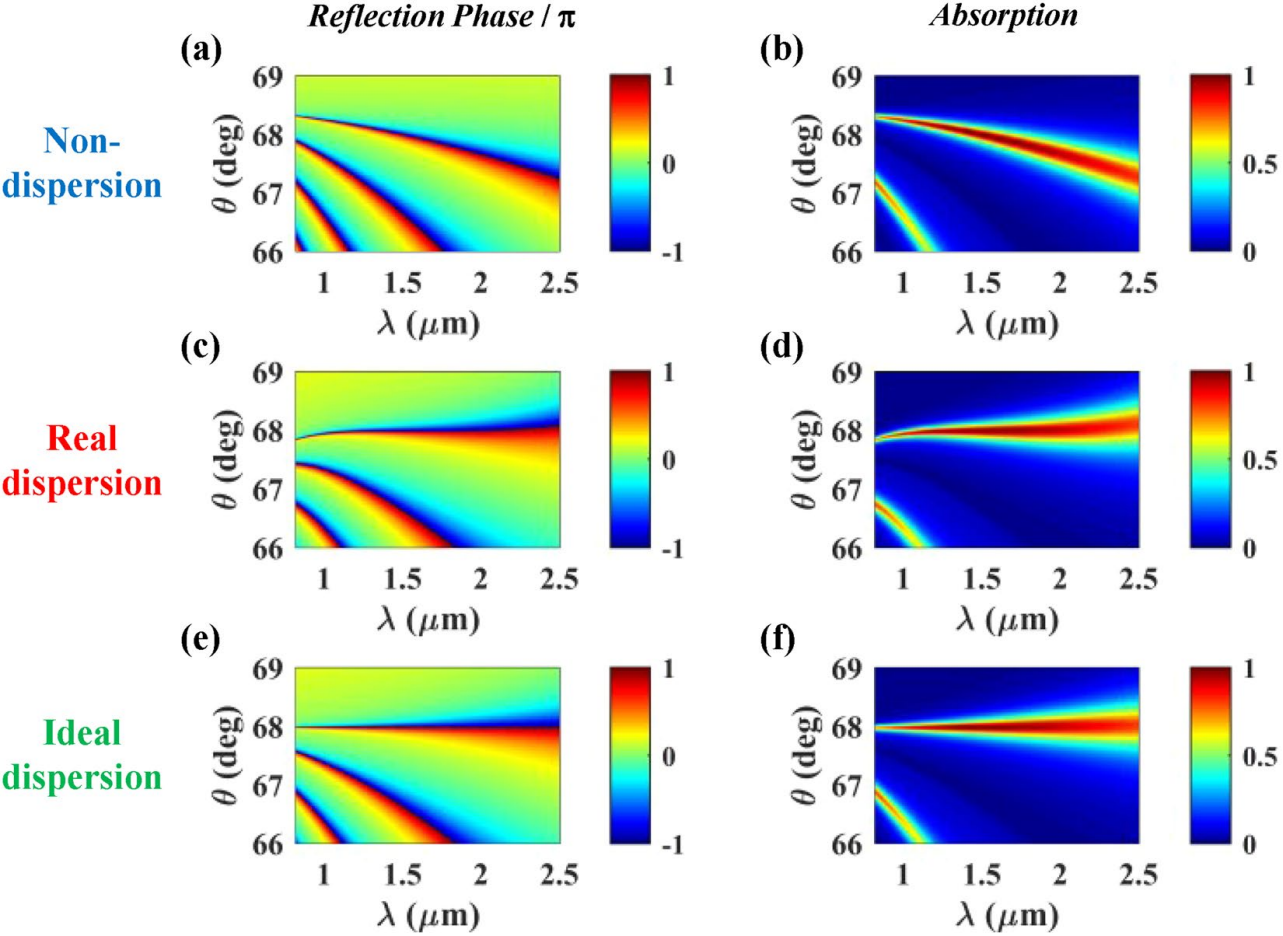
Reflection phase of the lossless structure (left panel, without graphene) and absorption (right panel, with graphene) as a function of wavelength and incidence angle for non-dispersion (top panel), real dispersion (middle panel), and ideal dispersion (bottom panel) of the prism (BK7) and the cavity layer (PDMS). All the remaining parameters are the same as those of Fig. 2. In the absorption analysis, the graphene layer was introduced in the middle of the cavity layer (*d*_A_ = 0.50*d*_2_).

**Figure 4:**
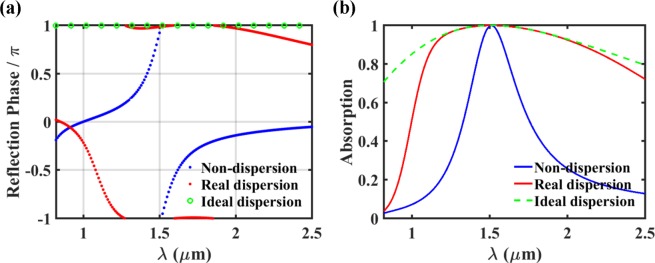
(**a**) Reflection phase and (**b**) absorption as a function of wavelength at optimal condition (*d*_2_ = 6.4 μm, *θ* = 67.98 deg) for non-dispersion (blue curves), real dispersion (red curves), and ideal dispersion (green curves) of the prism (BK7) and cavity layer (PDMS). All the curves are extracted from Fig. 3.

Since the phase matching condition was derived from *r* = −1, the phase of the reflected wave in the lossless structure is supposed to show the same wavelength dependency of the resonance (or phase matching) condition. The left panels in Fig. 3 show the phase of the reflection as a function of wavelength and incidence angle for the cases of non-dispersion, real dispersion, and ideal dispersion of the prism and the cavity layer, where all the parameters are just the same as those of Fig. 2. As expected, for the ideal-dispersion case, the reflection phase is exactly π over the whole wavelength range of our interest at the optimal incidence angle of *θ* = 67.98 deg. For the real dispersion case, at *θ* = 67.98 deg, there exists a considerably wide wavelength range (~1.3 μm < *λ* < ~2.0 μm) where the reflection phase is ~π, corresponding to the wavelength-insensitive phase matching. On the contrary, for the non-dispersion case, the reflection phase shows strong wavelength dependence at any incidence angle as expected. These results are also plotted in Fig. 4(a) at *θ* = 67.98 deg for clear comparison.

We also calculated the absorption as a function of wavelength and incidence angle for the three types of material dispersion (the right panel in Fig. 3). It is assumed that monolayer graphene is located in the middle of the cavity layer, that is, *d*_A_ = 0.5*d*_2_. Roughly speaking, the absorption peaks (including the perfect absorption at *λ* = 1.51 μm) exist along the loci of the resonance conditions with the reflection phase of π. So, the wavelength dependencies of absorption and reflection phase are identical. For the real and the ideal dispersion cases (Fig. 3(d,f)), at *θ* = 67.98 deg, there are the almost and the perfectly flat absorption peak branches, respectively. On the contrary, the absorption peak wavelength shows strong dependence on the incidence angle for the non-dispersion case (Fig. 3(b)). As seen in Fig. 4(b), in details, very high absorption value of A > 99% is maintained over ~1.37 μm < *λ* < ~1.67 μm (∆*λ* = ~300 nm) for the real dispersion case, while over ~1.492 μm < *λ* < ~1.534 μm (∆*λ* = ~42 nm) for the non-dispersion case. It is noted that the high absorption occurs over a part of the wavelength range where the reflection phase is ~π. These results indicate that it is possible to obtain the ultra-wideband absorption based on wavelength-insensitive phase matching. However, as checked from the ideal dispersion case in Fig. 4(b), the perfect absorption is obtained only at *λ* = 1.51 μm although the resonance condition is satisfied over the whole wavelength range of our interest. This is because the critical coupling condition (*γ*_loss_ = *γ*_leak_) is not satisfied over the wide wavelength range. Since both the loss and the leakage rates depend on wavelength, satisfying the critical coupling condition over the wide wavelength range is quite challenging, the effort on which is not exerted in this work. Nonetheless, only with the phase matching over the wide wavelength range, 99% absorption bandwidth of ~300 nm has been achieved. The FWHM of the designed perfect absorber appears to be larger than 1.5 μm. Due to the limit on the wavelength range where optical constants of the materials are available, the exact estimation of the FWHM could not be conducted.

**Figure 5:**
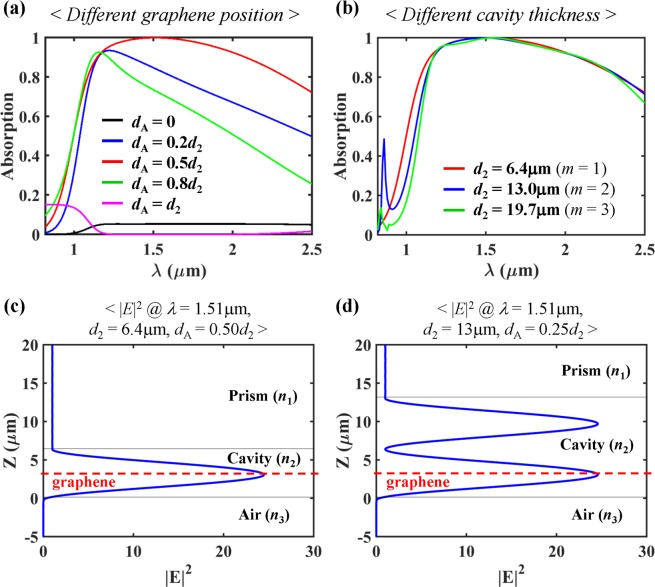
(**a**) Absorption spectra for different graphene position (*d*_A_) at *d*_2_ = 6.4 μm. (**b**) Absorption spectra for different cavity thickness with each optimal *d*_A_ (in details, *d*_A_ = 0.50*d*_2_ for *d*_2_ = 6.4 μm, *d*_A_ = 0.25*d*_2_ for *d*_2_ = 13.0 μm, and *d*_A_ = 0.50*d*_2_ for *d*_2_ = 19.7 μm). Also, spatial distributions of the normalized total electric field intensity at two perfect absorption conditions in (**b**) are plotted: (**c**) at (*λ* = 1.51 μm, *d*_2_ = 6.4 μm, *d*_A_ = 0.50*d*_2_), and (**d**) at (*λ* = 1.51 μm, *d*_2_ = 13 μm, *d*_A_ = 0.25*d*_2_). All the calculations are conducted for the real dispersion of the prism (BK7) and the cavity layer (PDMS), and the optimal incidence angle (*θ* = 67.98 deg).

In order to achieve perfect absorption at a specific wavelength within the phase matching wavelength range, the position of the graphene layer should be chosen carefully. Due to the electric fields with the standing wave pattern in the cavity layer, the loss rate can be controlled by the graphene position for a given absorber structure with a specific leakage rate, resulting in a change of the absorption spectra^16^. In our designed absorber structure of the real material dispersions with monolayer graphene, the optimal graphene position is *d*_A_ = 0.50*d*_2_ for *d*_2_ = 6.4 μm and *θ* = 67.98 deg, as demonstrated in Fig. 5(a). The spatial distribution of electric field intensity (|*E*|^2^) at the perfect absorption wavelength (*λ* = 1.51 μm) is plotted in Fig. 5(c). One can see that the perfect absorption is attributed to the 1^st^-order resonance (*m* = 1). Interestingly, the location of graphene (*d*_A_ = 0.50*d*_2_) almost coincides with the field maximum position (~0.50*d*_2_). But, in general, this is not necessary for the critical coupling. For example, for an another absorber structure (*d*_A_ = 0.35*d*_2_, *d*_2_ = 6.42 μm, *θ* = 67.98 deg) which provides A > 99% over ~1.26 μm < *λ* < ~1.54 μm (∆*λ* = ~280 nm), the graphene layer is quite far from the field maximum position (~0.50*d*_2_) at the perfect absorption wavelength (*λ* = 1.38 μm) (Supplementary Information). So, the location of the graphene layer should be chosen to satisfy the critical coupling condition, that is, to balance the loss rate to the leakage rate at a specific wavelength.

We additionally investigated the absorption performance for three different optimal values of *d*_2_ which correspond to *m* = 1, 2, 3 in the phase matching condition of (2*δ*_2_ − *ϕ* = 2π*m*) when *θ* = 67.98 deg, which is shown in Fig. 5(b). Due to different electric field profiles in the cavity layers of different thicknesses^16^, the different optimal *d*_A_ is chosen: *d*_A_ = 0.50*d*_2_ for *d*_2_ = 6.4 μm, *d*_A_ = 0.25*d*_2_ for *d*_2_ = 13.0 μm, and *d*_A_ = 0.50*d*_2_ for *d*_2_ = 19.7 μm. Figure 5(d) shows |*E*|^2^ at perfect absorption wavelength (*λ* = 1.51 μm) for the absorber structure of *d*_2_ = 13 μm, *d*_A_ = 0.25*d*_2_, which confirms that the perfect absorption is attributed to the 2^nd^-order resonance (*m* = 2). Despite of different *d*_2_, the ultra-broadband absorption is still sustained because all the absorber structures sustain the wavelength-insensitive phase matching over a wide wavelength range and the proper critical coupling condition at *λ* = 1.51 μm.

## Discussion

In this work, the ultra-wideband absorption based on the wavelength-insensitive phase matching has been investigated for the proposed perfect absorber of the Prism-Cavity-Air configuration with monolayer graphene embedded in the cavity. It is noteworthy that the key consideration is the proper combination of material dispersions of the prism and the cavity layer because it determines the optimal incidence angle, cavity thickness, and wavelength range for the wavelength-insensitive phase matching. As discussed earlier, the requirement on the material dispersions for the wavelength-insensitive phase matching over the wavelength range of our interest is summarized as *n*_01_ > *n*_02_, ${n}_{02}^{2}/{n}_{01}^{2} > ({A}_{32}/{\lambda }_{32}^{2})/({A}_{31}/{\lambda }_{31}^{2})$ and (*λ*_11_, *λ*_21_ *λ*_12_, *λ*_22_) ≪ *λ* ≪ (*λ*_31_, *λ*_32_).

**Figure 6:**
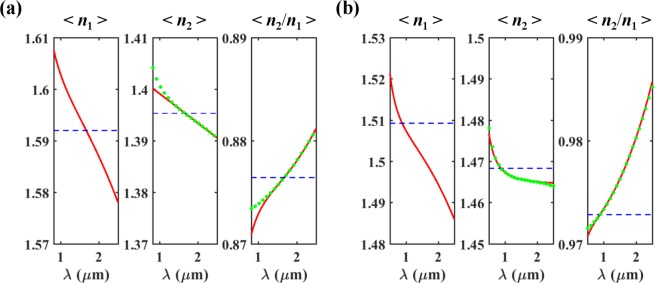
The material dispersion of (**a**) F2 glass (*n*_1_), PDMS (*n*_2_), the ratio (*n*_2_/*n*_1_) between them over the wavelength range of *λ* = 0.82~2.50 μm, and (**b**) BK7 glass (*n*_1_), Silica (*n*_2_), the ratio (*n*_2_/*n*_1_) between them over the wavelength range of *λ* = 0.50~2.50 μm. The blue, red, and green curves represent the cases of non-dispersion, real dispersion, and ideal dispersion, respectively.

**Figure 7:**
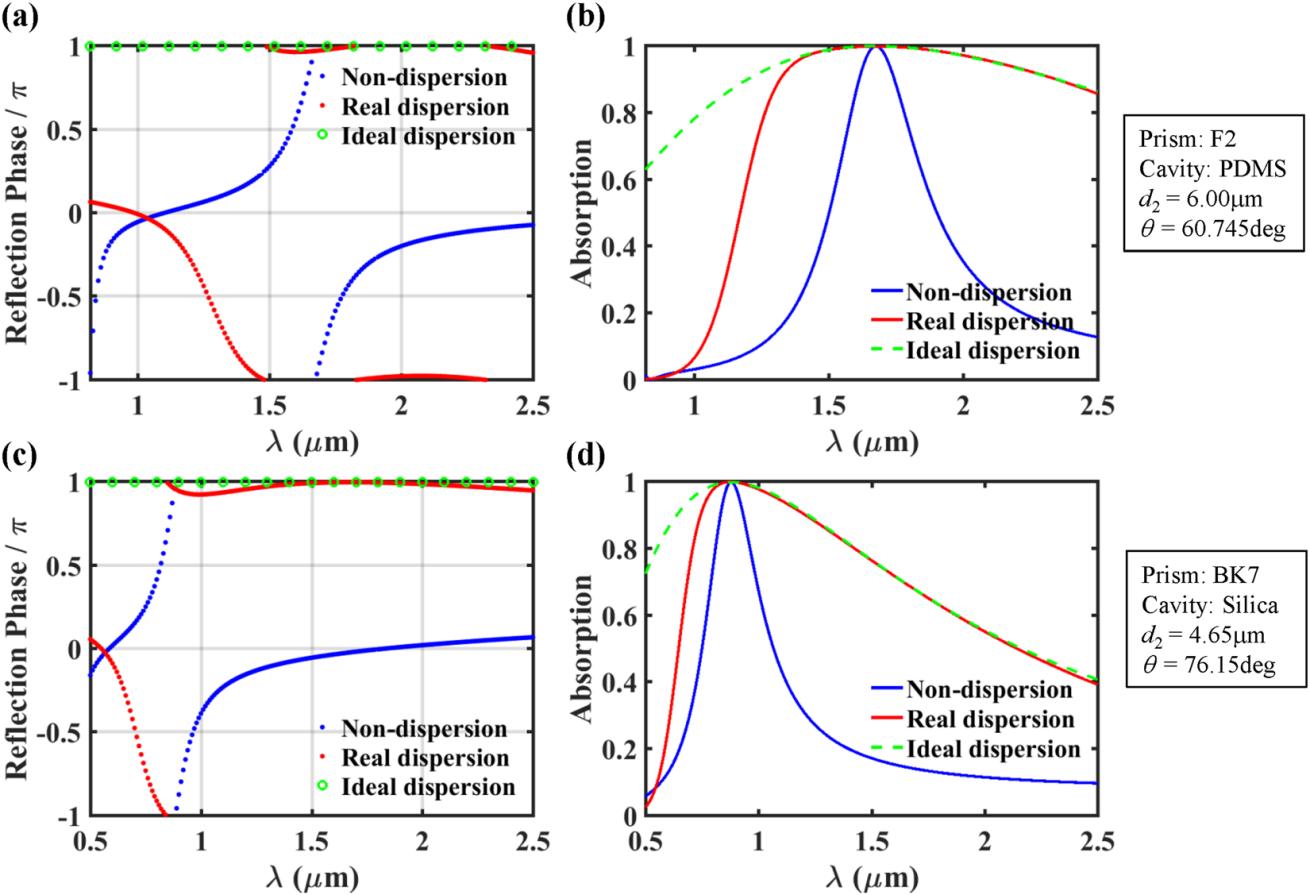
Reflection phase and absorption as a function of wavelength at each optimal condition for non-dispersion, real dispersion, and ideal dispersion when different material combinations of F2 & PDMS (top panel) and BK7 & Silica (bottom panel) are used. All the absorption analysis are for *d*_A_ = 0.50*d*_2_.

Practically, there can be various material combinations that meet the requirement. As a representative example of the prism and the cavity layer, BK7 and PDMS were assumed in the previous sections. It is also possible to obtain the ultra-wideband absorption based on the wavelength-insensitive phase matching with another material combinations, such as F2 & PDMS and BK7 & Silica. The real dispersions of the selected materials meet the requirement over the whole wavelength range of our interest, as verified in Fig. 6 ^31,32,35,36^. For the non-dispersion, real dispersion, and ideal dispersion cases, the reflection phase and absorption as a function of wavelength are calculated in the same way as the case of BK7 & PDMS. As expected, there exist the wide wavelength ranges with the reflection phase of ~π for the lossless structures (without graphene) of both the real and the ideal cavity material dispersions for the respective real prism materials, and much improved absorption bandwidth have been achieved compared to the non-dispersion cases (Fig. 7).

So far, absorption performance only for TE polarization is considered. Obviously, for TM polarization, the wavelength-insensitive phase matching condition by the inherent material dispersions of dielectric layers is identical to that for TE polarization. However, perfect absorption of ultra-wide bandwidth cannot be obtained for both polarizations simultaneously because a loss rate is sensitive to polarization. Owing to the very large magnitude of the permittivity of graphene (for example, above 16 at *λ* = 1.51 μm), for TM polarization, the dominant surface-normal electric field intensity in graphene and the resultant loss rate are very low^12,16^. For the material combinations considered here, multi-layer graphene should be used to achieve graphene perfect absorber of ultra-wide bandwidth for TM polarization (Supplementary Information).

## Methods

To numerically investigate and analyze the reflection phase and absorption properties in the proposed perfect absorber, we used the TMM^33,34^. In all our calculations, the complex permittivity of graphene (*ε*_*g*_) was calculated using Kubo formulation based on the local random phase approximation for various *E*_*f*_, assuming graphene thickness of 0.34 nm, Fermi velocity of 10^6^ m/s, and mobility of 0.5 m^2^/Vs^29,30^.

## Supplementary information

Supplementary information
