# Author Correction: Graphene perfect absorber of ultra-wide bandwidth based on wavelength-insensitive phase matching in prism coupling

**DOI:** 10.1038/s41598-019-55235-2

**Published:** 2019-12-05

**Authors:** Sangjun Lee, Hyungjun Heo, Sangin Kim

**Affiliations:** Department of Electrical and Computer Engineering, Ajou University, Suwon, South Korea

Correction to: *Scientific Reports* 10.1038/s41598-019-48501-w, published online 19 August 2019

A supplementary information containing Figures S1 and S2, and equations S1-S9, was omitted from the original version of this Article. This has been corrected in the HTML and PDF version of the Article.

