# Supplementary material for: Graphene perfect absorber of ultra-wide bandwidth based on wavelength-insensitive phase matching in prism coupling
Source: Sci Rep. 2019 Aug 19;9:11967. doi: 10.1038/s41598-019-48501-w (PMC6700108; doi:10.1038/s41598-019-48501-w)
Supplement: Supplementary file 1 — Supplementary information [file 41598_2019_48501_MOESM1_ESM.pdf]

< Supplementary Information >

Title: "Graphene perfect absorber of ultra-wide bandwidth based on wavelength-insensitive phase matching in prism coupling"

Sangjun Lee, Hyungjun Heo & Sangin Kim*

*Corresponding Author: E-mail:sangin@ajou.ac.kr

Department of Electrical and Computer Engineering, Ajou University, Suwon, South Korea

1. Reflection coefficient (r = -1) at the resonance condition

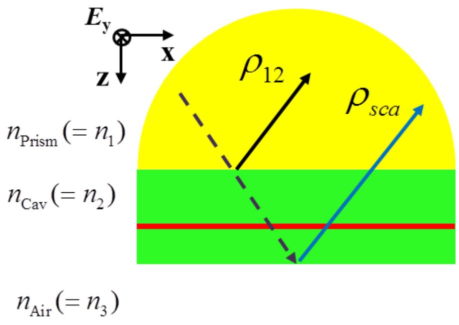

Figure S1. Theoretical model of the proposed graphene perfect absorber composed of Prism(n_1)-Cavity(n_2)-Air(n_3), where monolayer graphene is embedded in the cavity and $n_1 > n_2 > n_3 = 1$. The red thin layer represents the monolayer graphene of 0.34 nm thickness as an absorbing medium. ρ_{12} is Fresnel coefficients of the reflection at the prism-cavity interface, and ρ_{scatt} is the scattered waves via the multiple reflections from the other interface, assuming that the amplitude of the incidence wave (black dashed line) is unity.

We provide additional theoretical derivations to show that the reflection phase is approximately π at the resonance condition (or wavelength) in the proposed graphene perfect absorber based on prism coupling (Fig. S1). Because the graphene is too thin (0.34 nm), the graphene does not almost affect the resonance condition. So, the reflection phase of the dielectric multilayer structures (prism-cavity-air) without graphene is assumed to be the same as that of the perfect absorption condition [1, 2]. The perfect absorption occurs when the waves reflected from the prism-cavity interface (ρ_{12}) destructively interfere with the scattered waves via the multiple reflection from the other interface (ρ_{scatt}), assuming that the amplitude of the incidence wave is 1. This means that the reflection coefficient can be given by

$$r = \rho_{12} + \rho_{scatt} = 0 \quad \text{(S1)}$$

and thus, the amplitude and phase information is as follows.

$$|\rho_{12}| = |\rho_{scatt}|, \ \angle\rho_{12} = \angle\rho_{scatt} + \pi \quad \text{(S2)}$$

If the graphene layer is removed, | r | = -1 due to total internal reflection (TIR), and

$$|\rho_{12}| \neq |\rho_{scatt}|, \ \angle\rho_{12} = \angle\rho_{scatt} + \pi \quad \text{(S3)}$$

So, we obtain

$$r = |\rho_{12}| e^{j0} + |\rho_{scatt}| e^{j\pi} = |\rho_{12}| - |\rho_{scatt}| \quad \text{(S4)}$$

As a result, considering that $|r|$ = -1, and $|\rho_{12}|$ is small enough compared to $|\rho_{\text{scatt}}|$,

$$r = -1 \quad \text{(S5)}$$

For a given material combination of the prism and the cavity layer, the resonance conditions of r = -1 are determined by the cavity thickness and incidence angle.

2. Reflection coefficient based on TMM

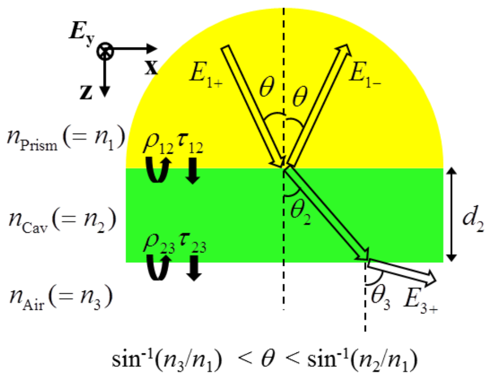

Figure S2. Schematic of the dielectric multilayer structures (prism-cavity-air) without a graphene layer, where $n_1 > n_2 > n_3 = 1$.

When the graphene layer is removed, the reflection coefficient of the proposed absorber structure (Fig. S2) can be derived by the transfer matrix method (TMM) [3, 4]. We considered transverse electric (TE, or s-polarized) wave illumination from the prism with an incidence angle of θ, whose electric field is perpendicular to the incidence plane. In general, the reflection or transmission coefficients can be calculated

with the TMM, in which electric fields in one position can be related to those in other positions through a transfer matrix, M. For the considered multilayer structure, total transfer matrix relating the incoming (E_{1+}) and outgoing (E_{1-}) components at the prism-cavity interface, and the incoming (E_{3-}) and outgoing (E_{3+}) components at the cavity-air interface is written as

$$\begin{bmatrix} E_{1+} \\ E_{1-} \end{bmatrix} = M \begin{bmatrix} E_{3+} \\ E_{3-} \end{bmatrix}, \qquad \text{(S6)}$$

where

$$M = \frac{1}{\tau_{12}} \begin{bmatrix} 1 & \rho_{12} \\ \rho_{12} & 1 \end{bmatrix} \cdot \begin{bmatrix} e^{j\delta_2} & 0 \\ 0 & e^{-j\delta_2} \end{bmatrix} \cdot \frac{1}{\tau_{23}} \begin{bmatrix} 1 & \rho_{23} \\ \rho_{23} & 1 \end{bmatrix}, \qquad \text{(S7)}$$

The phase retardation experienced by the wave during a round trip in the cavity layer is given by $2\delta_2 = 4\pi d_2 n_2 \cos\theta_2/\lambda$, where $n_2\cos\theta_2 = sqrt(n_2 \cdot n_2 - n_1 \cdot n_1 \cdot \sin\theta \cdot \sin\theta)$. Fresnel coefficients of the reflection (ρ_{12}, ρ_{23}) and transmission (τ_{12}, τ_{23}) at the prism-cavity and the cavity-air interfaces are given by

$$\begin{cases} \rho_{12} = \dfrac{n_1 \cos\theta - n_2 \cos\theta_2}{n_1 \cos\theta + n_2 \cos\theta_2} \\ \rho_{23} = \dfrac{n_2 \cos\theta_2 - n_3 \cos\theta_3}{n_2 \cos\theta_2 + n_3 \cos\theta_3} \end{cases}, \qquad \text{(S8)}$$

$$\begin{cases} \tau_{12} = \dfrac{2n_1 \cos\theta}{n_1 \cos\theta + n_2 \cos\theta_2} \\ \tau_{23} = \dfrac{2n_2 \cos\theta_2}{n_2 \cos\theta_2 + n_3 \cos\theta_3} \end{cases}, \qquad \text{(S9)}$$

Assuming there is no incoming wave from air ($E_{3-} = 0$), by rearranging Eq. (S6), we obtain

$$\begin{bmatrix} E_{1+} \\ E_{1-} \end{bmatrix} = \frac{E_{3+}}{\tau_{12}\tau_{23}} \begin{bmatrix} e^{j\delta_2} + \rho_{12}\rho_{23} e^{-j\delta_2} \\ \rho_{12} e^{j\delta_2} + \rho_{23} e^{-j\delta_2} \end{bmatrix}, \qquad \text{(S10)}$$

and thus, reflection coefficient (r) is given by

$$r = \frac{E_{1-}}{E_{1+}} = \frac{\rho_{12} + \rho_{23}\, e^{-j2\delta_2}}{1 + \rho_{12}\rho_{23}\, e^{-j2\delta_2}}, \qquad \text{(S11)}$$

In particular, at the resonance condition of r = -1, Eq. (S11) is rearranged as follows.

$$e^{j2\delta_2} = -\rho_{23} \qquad \text{(S12)}$$

3. Ultra-broadband absorption for different graphene positions

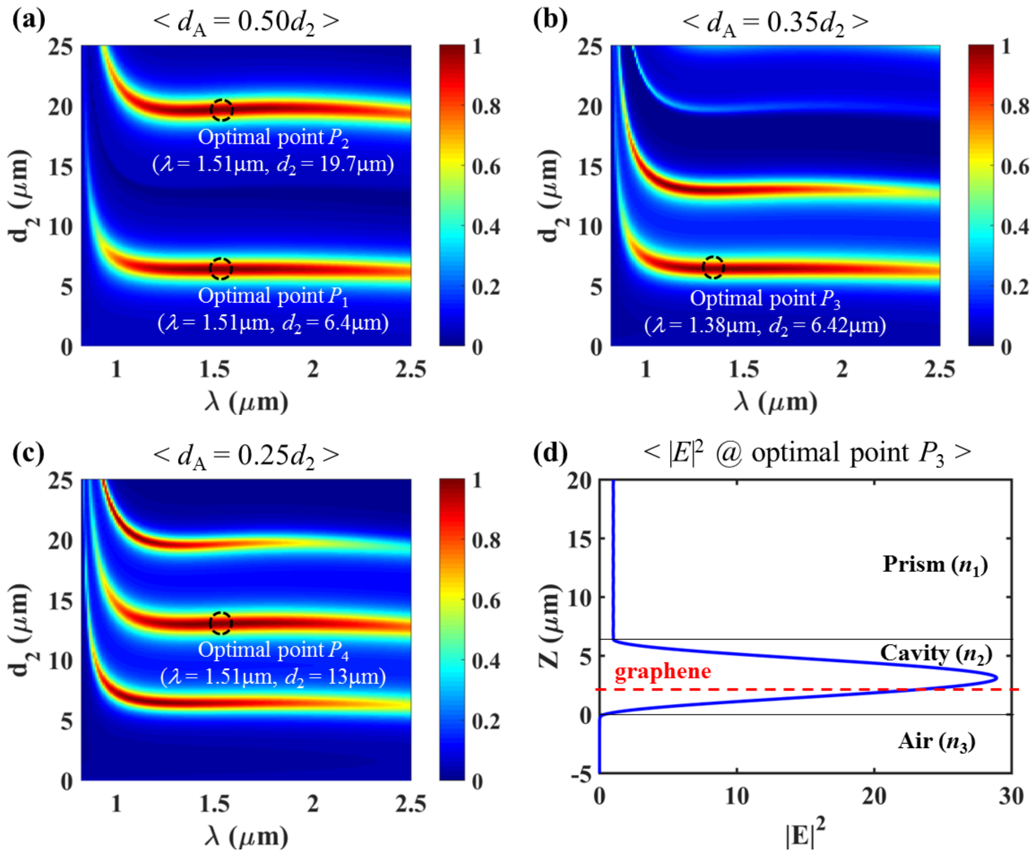

Figure S3. Absorption maps as a function of wavelength and cavity thickness for (a) d_A = 0.50d_2, (b) d_A = 0.35d_2, and (c) d_A = 0.25d_2. Also, $|E|^2$ at optimal point P_3 (d_A = 0.35d_2, d_2 = 6.42μm, λ = 1.38μm) is plotted in (d). All the calculations are conducted for the real dispersion of the prism (BK7) and the cavity layer (PDMS), and the optimal incidence angle (θ = 67.98deg).

When the relative position of graphene layer embedded in the cavity changes, the optimal (perfect) absorption wavelength changes so as to satisfy critical coupling condition, considering that wavelength-insensitive phase matching is not almost affected by the graphene layer. Actually, analysis for d_A = 0.50d_2 and d_A = 0.25d_2 are already discussed in the manuscript: in detail, the three curves in Fig 5(b) correspond to cross-section at optimal d_2 in Fig. S3(a) and Fig. S3(c). Fig. S3(b) shows the absorption map for d_A = 0.35d_2. The perfect absorption occurs at not λ = 1.51μm but λ = 1.38μm (indicated by optimal point P_3). Despite of different graphene positions, the similar ultra-broadband absorption can be sill sustained, as estimated from 'flat' absorption peak branches. In details, A > 99% over ~1.26μm < λ < ~1.54μm ($\Delta\lambda$ = ~280nm) for absorber structure of d_A = 0.35d_2, d_2 = 6.42μm, while A > 99% over ~1.37μm < λ < ~1.67μm ($\Delta\lambda$ = ~300nm) for absorber structure of d_A = 0.50d_2, d_2 = 6.4μm. Fig. S3(d) shows the spatial distribution

of electric field intensity ($|E|^2$) at optimal point P_3. The critical coupling condition can be satisfied although the graphene layer ($d_A = 0.35d_2$) is quite far from the field maximum position (~$0.50d_2$).

4. Absorption performance for TM polarization

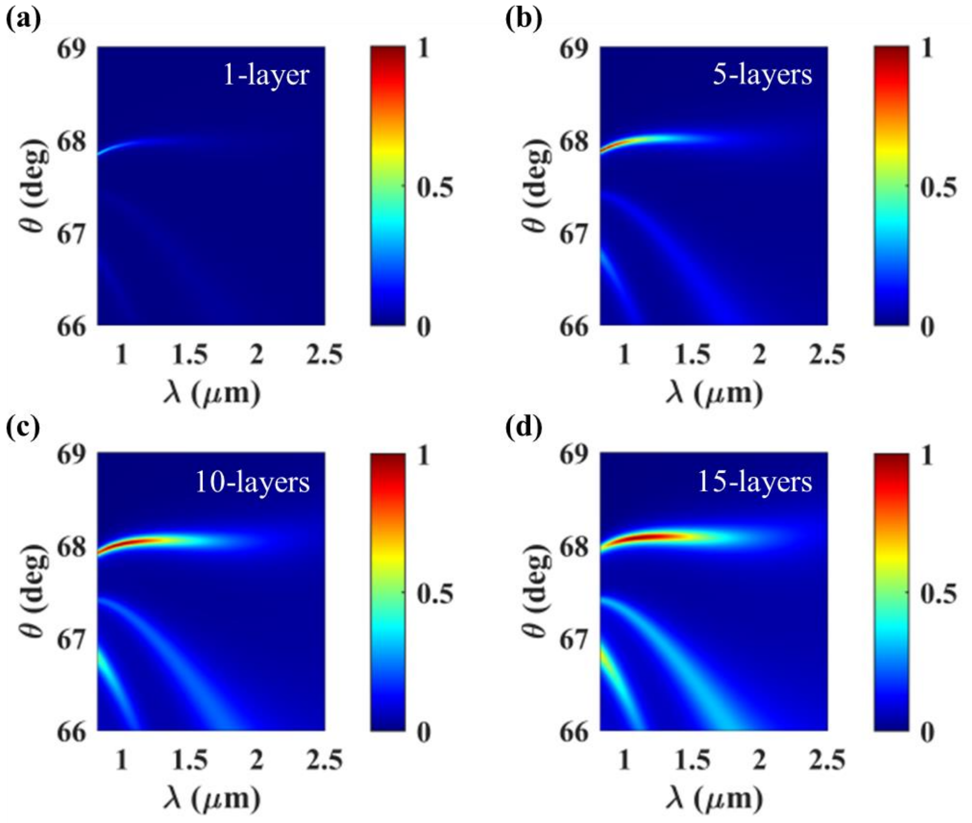

Figure S4. For TM polarization, absorption maps as a function of wavelength and incidence angle for (a) 1-layer graphene, (b) 5-layers graphene, (c) 10-layers graphene, and (d) 15-layers graphene. All the calculations are conducted for the real dispersion of the prism (BK7) and the cavity layer (PDMS), and the optimal incidence angle ($\theta = 67.98$deg).

Obviously, for TM polarization, the condition of wavelength-insensitive phase matching by the inherent material dispersion of dielectric layers is identical to that of TE polarization [1, 5], ignoring birefringence of materials. However, perfect (or ultra-broadband) absorption cannot be obtained for both polarizations simultaneously because a loss rate is sensitive to polarization. Boundary condition of electromagnetic fields, owing to the very large absolute permittivity of graphene (for example, above 16 at $\lambda = 1.51\mu$m) compared

to cavity permittivity, for TM polarization, the dominant surface-normal electric field intensity in graphene and resultant loss rate are very low, as shown in Fig. S4(a). In material combination considered here, the multi-layer graphene should be necessarily applied to achieve graphene perfect absorber of ultra-wide bandwidth. As seen in Fig. S4(b-d), regardless of the number of graphene layer, absorption peak branches follow the loci of the resonance conditions with the reflection phase of π.

References for Supplementary materials:

[Ref. S1] Lee, S. *et al*. Practical perfect absorption in monolayer graphene by prism coupling. *IEEE Photon. J*. **9**, 2700810 (2017).

[Ref. S2] Pirruccio, G. *et al*. Coherent and Broadband Enhanced Optical Absorption in Graphene. *ACS Nano*, **7**(6), 4810-4817 (2013).

[Ref. S3] Zhan, T. *et al*. Transfer matrix method for optics in graphene layers. *J. Phys.: Condens. Matter*. **25**, 215301 (2013).

[Ref. S4] Yeh, P. Optical Waves in Layered Media (Wiley, 2005).

[Ref. S5] Lee, S. *et al*. Angle- and position-insensitive electrically tunable absorption in graphene by epsilon-near-zero effect. *Opt. Express* **23**, 33350-33358 (2015).
